# Acoustic Field of a Linear Phased Array: A Simulation Study of Ultrasonic Circular Tube Material

**DOI:** 10.3390/s19102352

**Published:** 2019-05-22

**Authors:** Zhitao Xiao, Yongmin Guo, Lei Geng, Jun Wu, Fang Zhang, Wen Wang, Yanbei Liu

**Affiliations:** 1 School of Electronics and Information Engineering, Tianjin Polytechnic University, Tianjin 300380, China; xiaozhitao@tjpu.edu.cn (Z.X.); wujun@tjpu.edu.cn (J.W.); zhangfang@tjpu.edu.cn (F.Z.); wangwen@tjpu.edu.cn (W.W.); liuyanbei@tjpu.edu.cn (Y.L.); 2 School of Mechanical Engineering, Tianjin Polytechnic University, Tianjin 300380, China; 1620031051@stu.tjpu.edu.cn; 3 Tianjin Key Laboratory of Optoelectronic Detection Technology and System, Tianjin Polytechnic University, Tianjin 300387, China

**Keywords:** circular tube material, linear phased-array ultrasonic transducer, Multi-Gaussian beam model, tapered probe wedge, focused beam

## Abstract

As ultrasonic wave field radiated by an ultrasonic transducer influences the results of ultrasonic nondestructive testing, simulation and emulation are widely used in nondestructive testing. In this paper, a simulation study is proposed to detect defects in a circular tube material. Firstly, the ultrasonic propagation behavior was analyzed, and a formulation of the Multi-Gaussian beam model (MGB) based on a superposition of Gaussian beams is described. The expression of the acoustic field from a linear phased-array ultrasonic transducer in the condition of a convex interface on the circular tube material is proposed. Secondly, in order to make the tapered probe wedge better fit the curved circular tube material and carry out the ultrasonic inspection of the curved surface, it was necessary to pare the angle probe wedge. Finally, acoustic field simulations in a circular tube were carried out and analyzed. The simulation results indicated that the method of ultrasonic phased-array inspection is feasible in circular tube testing. Tube materials with different curvatures need different array element lengths and widths to get the desired focused beam.

## 1. Introduction

Tubular parts have been widely used in petroleum, natural gas, chemical industry, aerospace, ship, construction, and nuclear industry. These industries play an important role in national economy, national defense construction, and high-tech-related fields [1,2,3]. The quality of the materials used in tubular parts directly determines the operation performance and service life of the related equipment and has always demanded a rigorous nondestructive testing technology. With the rapid development of physics, material science, microelectronics, and computer technology, more than 70 kinds of different methods in nondestructive testing technology have been developed [4,5,6,7], mostly based on penetration, magnetic powder, radiation, eddy current, and ultrasonic testing. Among the various nondestructive testing techniques, the ultrasonic nondestructive testing technology has good technical characteristics of sound beam controllability, imaging diversification, and detection automation and becomes an important means of quality monitoring of circular tube materials. It has outstanding performances in flaw detections for weld joint, composite material, and various components in the aerospace or nuclear industry [8,9,10,11].

Phased-array ultrasonic imaging has developed as a result of the improvement of electronic information technology and has made great progress in the medical field. However, in the industrial field, although, to a certain extent, the problems existing in conventional ultrasonic have been solved, it still faces many challenges. These include the fact that the object structure and the material of industrial ultrasonic imaging are very different, the large amount of industrial phased-array ultrasonic imaging data, the complex hardware architecture [12], the bad detection environment, the problem that the imaging results are easily disturbed by the scene. Circular tube material online phased-array ultrasonic imaging is an important component of industrial phased-array ultrasonic imaging, which, in addition to suffer from the common imaging problems indicated above, presents an additional issue related to the circular tube materials, since the theoretical basis of tubular detection is still not well developed. Considering this background, a study method of the acoustic field from a linear phased-array ultrasonic transducer for circular tube material is proposed.

**Figure 1:**
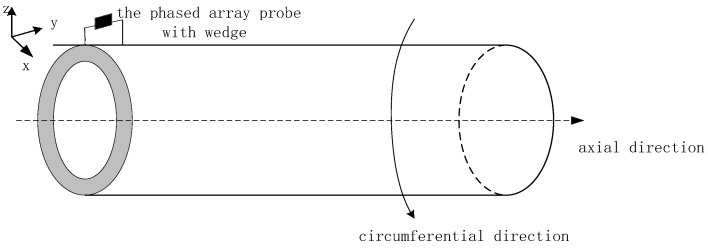
Schematic diagram of the scanning tube material path with phased-array ultrasonic transducer, with the transducer placed on the convex circular tube interface.

In actual testing, wedges are always used to couple the array to the tested material. In the inspection of circular tube material, according to its convex shape, the probe wedge needs to fit different inner diameters. So, acoustic field simulations are carried out under the convex cylindrical interface between the wedge and surface of the circular tube material. In the present study, a phased-array ultrasonic transducer was placed on the surface of the material, scanning along the circumferential direction of the material, as shown in Figure 1. The phased-array transducer was a linear transducer.

In order to simulate the acoustic field, various models have been developed [13,14,15,16]. Song and Kim have calculated a 3D radiation beam model propagated through a planar interface from ultrasonic phased-array transducers based on the Rayleigh–Sommerfeld integral [17]. However, the Rayleigh–Sommerfeld integral requires lengthy calculations, and the model is not appropriate for cases in which focusing is performed on curved interfaces, such as circular tube materials. The simulation method was based on the multivariate Gaussian beam model (MGB), which can effectively evaluate the effects of complex geometry and material on the sound field of the probe and is able to get the analytical expression of the sound field. So, its computation efficiency is high. MGB can be used to simulate the piston, the rectangular and phased-array probe emission sound fields, the acoustic field in heterogeneous anisotropic media spreading on an irregular interface, and the waveform transformation in the process of transmission. With these advantages, the multi-Gaussian beam model has been widely used [18,19,20] in ultrasonic detection modeling and simulation. The MGB model for composites coupled with water was introduced into an automatic ultrasonic testing system [21]. Acoustic field simulations based on MGB in the condition of concave cylindrical interfaces were developed [22]. Up to now, however, no simulation and systematic investigation of the acoustic field characteristics from a phased-array ultrasonic transducer in the condition of convex circular tube interface have been performed.

In this paper, a simulation method based on MGB is proposed to detect the defects in a circular tube material. Firstly, the expression of the acoustic field in the condition of a convex interface was derived according to MGB and ray acoustics theory, and then acoustic field simulations in the circular tube material were carried out. In order to make the tapered probe wedge better fit the curved circular tube material, it was necessary to pare the angle probe wedge for ultrasonic inspection of the curved surface. Finally, the acoustic field distribution when the probe was placed in water and in the condition of a convex cylindrical interface between the probe with a tapered wedge and the circular tube material was obtained. The main contributions of our article include: (1) A complete model was established to detect the defects in circular tube material. The model was divided into two sub-models: the acoustic field simulation model based on MGB and the model to pare the angle probe wedge for ultrasonic inspection of curved surface. The two sub-models were then combined, so as to be applicable for the detection of internal defects in the circular tube material. (2) Acoustic field simulations were carried out in the circular tube material. The simulation results showed that, to obtain optimal focusing beams, the tube material with different curvatures needed elements with different sizes.

## 2. Acoustic Field Production by a Single Element of the Phased Array

**Figure 2:**
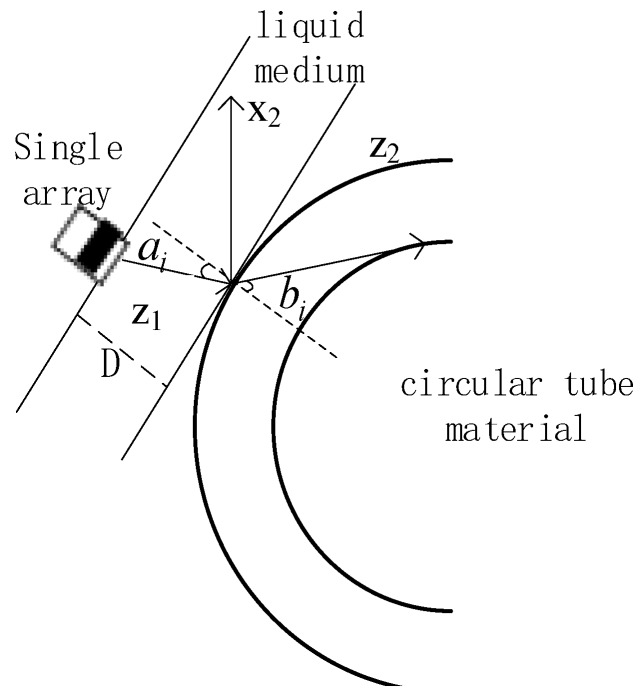
A multi-Gaussian beam propagates through the interface in the x–z plane: $a_{i}$ is the incidence angle of the longitudinal wave in the circular tube material, $b_{i}$ is the refraction angle of the longitudinal wave in the circular tube material.

The solid wedge is considered as an equivalent fluid medium because the liquid couplant between the wedge and the detected circular tube material cannot carry any shear stress. Thus, in our model, the wedge was perceived as liquid medium (V_1_) and the detected circular tube material was the solid medium (V_2_). That is to say, issues associated with a phased-array ultrasonic detection with a wedge can be simplified into liquid–solid interface problems. The transmission of a Multi-Gaussian beam through the interface in the x–z plane is shown in Figure 2.

### 2.1. Multi-Gaussian Beam Overlay Model in Liquid Medium

The Multi-Gaussian model is based on the theory of the paraxial approximation. MGB is the acoustic field produced by a circular piston source and can be calculated by super-position of a series of Gaussian beam functions [23]; the sound pressure expression of a probe in liquid can be written as:

(1) $$p\left(x_{1},y_{1},z_{1}\right)=\rho_{1}v_{1}\sum_{i=1}^{15}\frac{v_{o}A_{n}\exp(ik_{1}z_{1})}{1+(iB_{n}z_{1}/R_{i})}\exp\left(-\frac{B_{n}(x_{1}^{2}+y_{1}^{2})/r^{2}}{1+\left(iB_{n}z_{1}/R_{i}\right)}\right)$$

where $\rho_{1},v_{1}$ are the density and longitudinal wave velocity of the fluid medium, ${Α}_{n},{Β}_{n}$ are complex constants obtained by Wen and Breazeale, as shown in Table 1, r is the radius of the straight probe, $v_{0}$ is the velocity on the face of the transducer, $R_{i}=\frac{1}{2}k_{1}r^{2}$ is the Rayleigh distance, $k_{1}=\frac{2\pi }{\lambda }=\frac{w}{v_{1}}$ is the wave number in liquid.

A rectangular piston source can be expressed as the product of circular piston sources [23]. Thus, the acoustic field from a rectangular transducer can be calculated by superposition of two-dimensional Gaussian beams. The model is known as extended multivariate Gaussian beam model [25]. Generally speaking, in the case of far field and half-near field, the higher the number of superimposed Gaussian beams, the more accurate the simulation; the superposition of 15 sound beams is sufficiently accurate. In the tapered wedge in our study, the expression of the acoustic field from the nth single element can be written as:

(2) $$p_{n}\left(x_{1},y_{1},z_{1}\right)=\rho_{1}v_{1}\sum_{m=1}^{15}\sum_{n=1}^{15}\frac{v_{0}A_{n}A_{m}}{\sqrt{1+iB_{n}z_{1}/R_{1}}\sqrt{1+iB_{m}z_{1}/R_{2}}}\exp(ik_{1}z_{1})\exp\left[iw\left(\frac{1}{2}x^{T}M_{mn}(z_{1})x\right)\right]$$

where $R_{1}=\frac{1}{2}k_{1}a^{2}$, $R_{2}=\frac{1}{2}k_{1}b^{2}$, and 2a and 2b are the width and length of the rectangular element, respectively.

(3) $$\{\begin{array}{c} {\left[M_{mn}(z_{1})\right]}_{11}=\frac{{\left[M_{mn}\left(0\right)\right]}_{11}}{1+v_{1}z_{1}{\left[M_{mn}\left(0\right)\right]}_{11}}\\ {\left[M_{mn}(z_{1})\right]}_{22}=\frac{{\left[M_{mn}\left(0\right)\right]}_{22}}{1+v_{1}z_{1}{\left[M_{mn}\left(0\right)\right]}_{22}}\\ {\left[M_{mn}(z_{1})\right]}_{21}={\left[M_{mn}(z_{1})\right]}_{12}=0 \end{array}$$

For the matrix piston probe:

(4) $$\{\begin{array}{c} {\left[{Μ}_{mn}\left(0\right)\right]}_{11}=iB_{n}/v_{1}R_{1}\\ {\left[M_{mn}\left(0\right)\right]}_{22}=iB_{m}/v_{1}R_{2}\\ {\left[M_{mn}\left(0\right)\right]}_{21}={\left[M_{mn}\left(0\right)\right]}_{12}=0 \end{array}$$

According to Equations (2)–(4), (2) can be simplified as:

(5) $$\begin{array}{l} p_{n}\left(x_{1},y_{1},z_{1}\right)=\rho_{1}v_{1}\sum_{m=1}^{15}\sum_{n=1}^{15}\frac{v_{0}A_{n}A_{m}}{\sqrt{1+iB_{n}z_{1}/R_{1}}\sqrt{1+iB_{m}z_{1}/R_{2}}}\exp(ik_{1}z_{1})\exp(-\frac{B_{n}\frac{x_{1}^{2}}{a^{2}}}{1+iB_{n}z_{1}/R_{1}})\\ \times \exp(-\frac{B_{m}\frac{y_{1}^{2}}{b^{2}}}{1+iB_{m}z_{1}/R_{2}}) \end{array}$$

### 2.2. Acoustic Field in Solid Medium in the Condition of a Convex Cylindrical Interface

In the detected circular tube material, the acoustic field from a single rectangular element can also be calculated by superposition of two-dimensional Gaussian beams. Thus, the expression of the acoustic field from the nth single element in the solid medium can be written as [26]:

(6) $$\begin{array}{l} \frac{-iwu_{n}^{\alpha }(x_{2},y_{2},z_{2})}{v_{0}}=T_{12}^{\alpha :p}\sum_{m=1}^{15}\sum_{n=1}^{15}d^{\alpha }\frac{A_{n}}{1+iB_{m}D/R_{1}}\frac{A_{m}}{1+iB_{n}D/R_{2}}\\ \times \exp(ik_{1}D+ik_{2}^{\alpha }z_{2})\frac{\sqrt{\det G_{2}^{\alpha }\left(0\right)}}{\sqrt{\det G_{2}^{\alpha }(z_{2})}}\exp(\frac{ik_{1}x^{T}{\left[G_{2}^{\alpha }(z_{2})\right]}^{-1}x}{2}) \end{array}$$

where $u_{n}^{\alpha }$ is the displacement vector, $v_{0}$ is the velocity on the face of the transducer, $d^{\alpha }$ is polarization unit vector, $T_{12}^{\alpha :p}$ is the transmission coefficient under condition of liquid and solid interface, D is the propagation distance in the liquid medium, $k_{2}^{\alpha }=\frac{w}{v_{2}}$ is the wave number of longitudinal wave or shear wave in the solid medium, $x^{T}=[x_{2},y_{2}]$ is the position vector, $G_{2}^{\alpha }\left(0\right)$ and $G_{2}^{\alpha }(z_{2})$ are matrices in 2D and can be expressed as:

(7) $$\{\begin{array}{c} {\left[G_{2}^{\alpha }\left(0\right)\right]}_{11}=\frac{\frac{\cos b_{i}}{\cos a_{i}}{\left[G_{1}^{P}\left(D\right)\right]}_{11}}{\frac{\cos a_{i}}{\cos b_{i}}+\frac{\kappa_{1}\left(\cos a_{i}-\frac{v_{1}}{v_{2}^{\alpha }}\cos b_{i}\right)}{\cos a_{i}\cos b_{i}}{\left[G_{1}^{P}\left(D\right)\right]}_{11}}\\ {\left[G_{2}^{\alpha }\left(0\right)\right]}_{22}=\frac{{\left[G_{1}^{P}(D)\right]}_{22}}{1+\kappa_{2}(\cos a_{i}-\frac{v_{1}}{v_{2}^{\alpha }}\cos b_{i}){\left[G_{1}^{P}\left(D\right)\right]}_{22}}\\ {\;\;\;\;[G_{2}^{\alpha }\left(0\right)]}_{12}={\left[G_{2}^{\alpha }\left(0\right)\right]}_{21}=0\\ \;\;\;\;G_{2}^{\alpha }(z_{2})=G_{2}^{\alpha }\left(0\right)+\frac{v_{2}^{\alpha }}{v_{1}}z_{2}I \end{array}$$

where ${\left[G_{1}^{P}\left(D\right)\right]}_{11}=-\frac{iD_{1}}{B_{m}}+D,{\left[G_{1}^{P}\left(D\right)\right]}_{22}=-\frac{iD_{2}}{B_{j}}+D$, ${\left[G_{1}^{P}\left(D\right)\right]}_{12}={\left[G_{1}^{P}\left(D\right)\right]}_{21}=0$, $a_{i}$ is the incidence angle of the longitudinal wave in liquid medium, $b_{i}$ is the refraction angle in the solid medium, I is unit matrix in 2D, κ is the interface curvature of the circular tube material interface. In our present study, κ was a positive value because the boundary was raised against the liquid side from the solid side.

## 3. Acoustic Field from a Linear Phased-Array Transducer

An ultrasonic phased array pulses the individual probe elements at slightly different times according to different time delay rules, so that the individual wave fronts generated by each element in the array combine with each other to add or cancel energy in predictable ways that steer and shape the sound beam. So, the acoustic field from a linear phased-array transducer in liquid (tapered wedge) and solid (circular tube material) media can be expressed respectively as:

(8) $$p\left(x_{1},y_{1},z_{1}\right)=\sum_{n=1}^{N}p_{n}(x_{1},y_{1},z_{1})\exp(iwt_{n})$$

(9) $$\frac{-iwu^{\alpha }(x_{2},y_{2},z_{2})}{v_{0}}=\sum_{n=1}^{N}\frac{-iwu_{n}^{\alpha }(x_{2},y_{2},z_{2})}{v_{0}}\exp(iwt_{n})$$

where $t_{n}$ is the time delay of the nth element, N is the total number of elements.

### 3.1. Time Delays of Individual Elements

The linear phased-array ultrasonic probes are usually used with tapered wedges. Their characteristics are described below.

#### 3.1.1. Time Delay in Wedge

**Figure 3:**
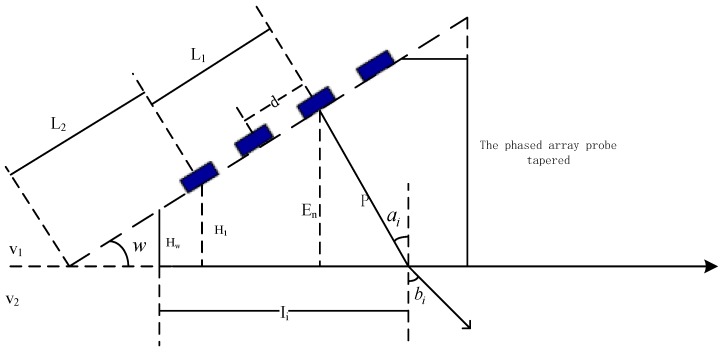
Calculation of the delay time of a phased-array probe wedge.

The time delay in a tapered wedge is the time required for ultrasonic propagation in the wedge, as shown in Figure 3. The calculation steps are as follows:

According to Snell’s law, the wedge internal incident angle $a_{i}$ of the corresponding angle of the refraction angle $b_{i}$ given by the specimen is calculated:

(10) $$a_{i}=\arcsin(\frac{v_{1}}{v_{2}}\sin b_{i})$$

The phased control probe midpoint (effective launching point) height $E_{n}$ is given by equation:

(11) $$E_{n}=(L_{1}+L_{2})\sin w=H_{1}+(n-1)d\sin w$$

The wave propagation distance in the wedge is P:

(12) $$P=\frac{E_{n}}{\cos a_{i}}$$

Finally, the delay time in the wedge can be written as:

(13) $$D_{w}=\frac{2P}{v_{1}}$$

where $H_{1}$ is the midpoint height of the first element, $H_{W}$ is the height of the back end of the wedge, $L_{1}$ is the distance between the midpoint of the first array and the ultrasonic incident point, $L_{{}_{2}}$ is the distance from the rear array to the horizontal line (the oblique contact surface), $v_{1}$ is the velocity of sound in the wedge, $v_{2}$ is the velocity of sound in the circular tube woven composite material specimen, d is the spacing between the centers of adjacent arrays.

**Figure 4:**
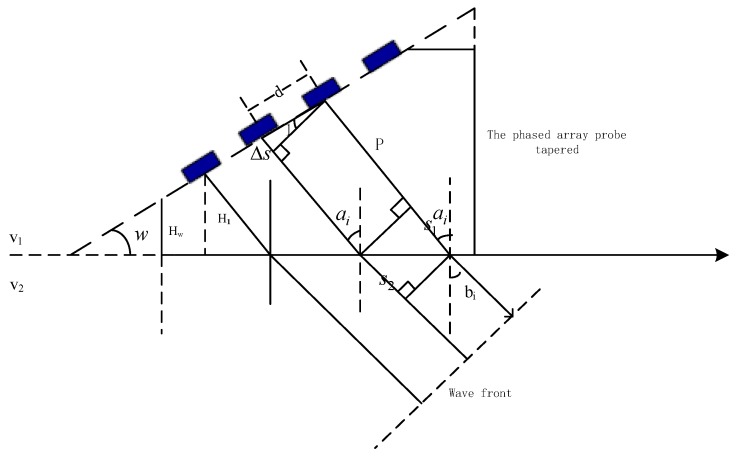
Steering beam from the linear phased-array transducer.

Time delays are calculated based on ray acoustics theory. Phased-array probes are used to transmit and receive ultrasonic beams [27]. The launch and time delay of each element were set up to stimulate one or several elements by using a phased-array instrument software in order to produce sound waves with controllable predetermined phases. The ultrasonic beam transmitted (or recived) by each array element produces a certain phased difference in the space sound field, the same phase will be superimposed, the opposite phase will cancel out, thus forming beam focusing or defection, etc. The steering beam is shown in Figure 4.

According to Snell’s law, the time of wave propagation trough the distance $s_{1}$ in the tapered wedge is the same as that through the distance $s_{2}$ in the circular tube material; Δs is the acoustic path difference between two adjacent elements; ∠1 is $a_{i}-w$. Thus, Δs can be written as:

(14) $$\Delta s=d\sin\left(a_{i}-w\right)$$

The delay time of the nth element relative to the first one can be written as:

(15) $$t_{n}=\frac{\left(n-1)d\sin(a_{i}-w\right)}{v_{1}}$$

#### 3.1.2. Time Delays for the Focusing Beam

**Figure 5:**
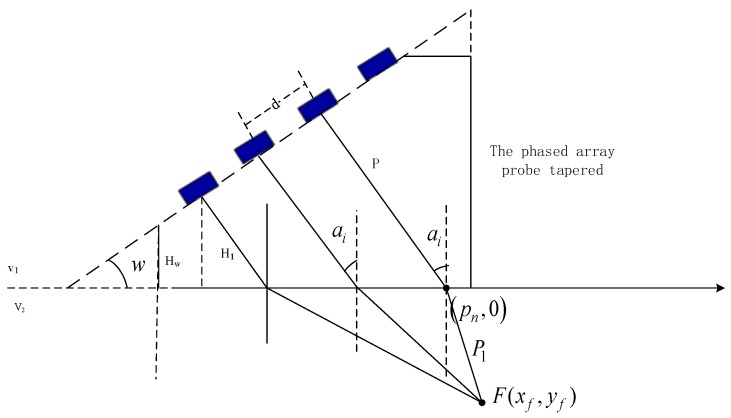
Focusing beam from the linear phased-array transducer in the tapered wedge.

The focusing beam from the linear phased-array transducer in the tapered wedge is shown in Figure 5.

From Figure 5, assume the focus point of the beam is $F(x_{f},y_{f})$, the intersection point of the beam generated by the nth element with the interface is $\left(p_{n},0\right)$, the wave propagation distance in the circular tube material is $P_{1}$, the delay time in the tapered wedge is $D_{w}=\frac{2P}{v_{1}}$. According to the geometric relationships, $P_{1}=\sqrt{y_{f}^{2}+{\left(x_{f}-p_{n}\right)}^{2}}$, P can be calculated by Equations (10)–(12). Then, the total time of wave propagation through the tapered wedge and circular tube material for the nth element can be written as:

(16) $$T_{n}=\frac{2P}{v_{1}}+\frac{2P_{1}}{v_{2}}$$

Assume the total propagation time of the first pulsed element is T, and the delay time of the nth element relative to the first one can be written as:

(17) $$t_{n}=T-T_{n}$$

## 4. Paring the Angle Probe Wedge for Ultrasonic Inspection of a Curved Surface

**Figure 6:**
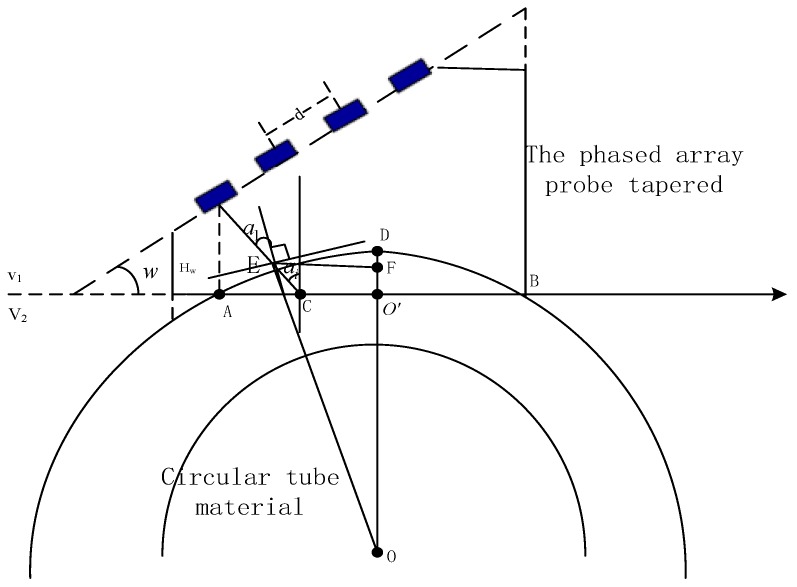
Schematic of the wedge after grinding.

The ultrasonic angle probe is used to detect material with a small radius curvature [28]. The refraction angle of the probe and the incident point are changed because the contact gap between the probe and the material surface is large, which causes a defect location error and affects detection sensitivity. In order to avoid this, we used the GB11345-89 steel weld manual ultrasonic flaw detection method and based the test on this condition: if the base of the wedge is not pared, the wedge length L should be such that $R>\frac{L^{2}}{4}$ (R is the material surface radius). Thus, it was necessary to modify the wedge. Figure 6 is the tapered wedge after the paring of the surface.

In Figure 6, $a_{1}=a_{i}+∠EOD$, E is the point of incidence after paring. Assuming Δa is the change value in the incident angle, $a_{1}=a_{i}+\Delta a$, h is the paring height, R is the outer diameter, DF≈0, then Δa=∠EOD, DO’ = DF + FO’ = FO’ = h, $O^{\prime }B=\sqrt{R^{2}-{\left(R-h\right)}^{2}}$. Generally speaking, the higher the paring height, the larger the contact surface between the probe and the material. However, if the contact surface is too large, the element can wear out, thus reducing the emission intensity of the probe. In order to avoid this, the paring height must be less than or equal to the interval of the height from the first element to the bottom of the tapered wedge. On the basis of experience, when the length of the probe contacts the material is 2/3 of the length of the probe, the stability can be guaranteed. That is to say, $2O^{\prime }B=2\sqrt{R^{2}-{\left(R-h\right)}^{2}}\geq \frac{2}{3}L$, $R-\sqrt{R^{2}-{\left(\frac{L}{3}\right)}^{2}}\leq h\leq 2H_{1}-H_{w}$.

## 5. Simulation Results and Discussion

Considering a longitudinal wave, acoustic field simulations were carried out in a circular tube material using the model described above. The material was steel, whose density is 7.8 × 10^3^ kg/m^3^, and the velocities in the wedge and in the material were 2730 m/s and 3230 m/s, respectively. The wedge was made of Plexiglas, whose density is 1.18 × 10^3^ kg/m^3^. The center frequency of the linear phased-array ultrasonic transducer was 5 MHZ, the element width 2a was 4 mm, the element length 2b was 13 mm, and the inter-spacing between the centers of adjacent elements d was 5.9 mm. The distance between the probe and the incident point was 17.1 mm. In this paper, we implemented the simulation method in MATLAB R2012a and windows 7.

**Figure 7:**
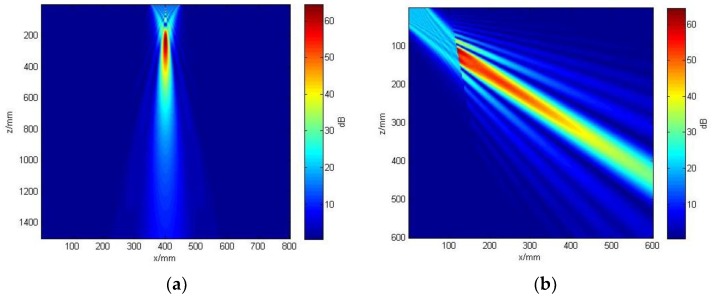
The acoustic field: (**a**) acoustic field of the probe in water; (**b**) acoustic field of the probe with a tapered wedge in the circular tube material in the condition of a convex cylindrical interface.

The acoustic field distribution of the beams is shown in Figure 7. Figure 7a is the acoustic field of the probe in water. Figure 7b is the acoustic field of the probe with a tapered wedge in the circular tube material in the condition of a convex cylindrical interface. It is shown that a linear phased-array ultrasonic transonic transducer can produce a routine focusing beam in the circular tube material in the condition of a convex cylindrical interface, and the energy is concentrated near the expected focal point. These results demonstrated that the method of phased-array inspection is valid for circular tube material testing.

There are two factors affecting the beam steering and focusing behaviors: one is the surface curvature of the circular tube material, the other is caused by the length and the width of the element. The effect of the surface curvature of the circular tube is discussed first.

**Figure 8:**
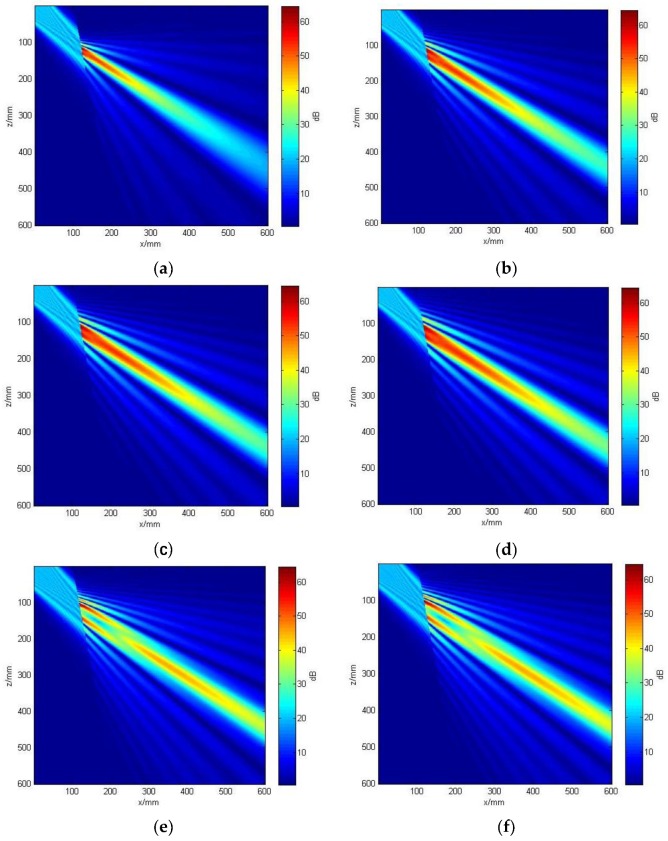
The acoustic field in materials with different curvatures: (**a**) acoustic field in material with a curvature of 1/0.35, with element width of 4 mm, and element length of 13 mm; (**b**) acoustic field in material with a curvature of 1/0.45, with element width of 4 mm, and element length of 13 mm; (**c**) acoustic field in material with a curvature of 1/0.5, with element width of 4mm, and element length of 13 mm; (**d**) acoustic field in material with a curvature of 1/0.6, with element width of 4 mm, and element length of 13 mm;(**e**) acoustic field in material with a curvature of 1/0.45 with element width of 5 mm, and element length of 15 mm; (**f**) acoustic field in material with a curvature of 1/0.5, with element width of 5 mm, and element length of 15 mm.

To see how the curvature parameters affect the characteristics of the propagating beam, we artificially changed the curvature parameters of the circular tube material in the case that the length and the width of the element were 13 mm and 4 mm, respectively. Clearly, something changed when the curvature was between the 1/0.6 and 1/0.35. Different diameters of the tube affected the focusing behavior of the beam because of their different surface curvature, as shown in Figure 8. The simulation results in Figure 8a–d show the effect of the tube with different curvatures on the incident beam. It is shown that the beam-focusing ability of the linear phased-array was changed by the curvature parameters of the circular tube material. When the length and the width of the element remained constant, as the curvature of the circular tube increased, the acoustic beams converged. As the curvature parameters decreased, the transmitted beam moved away from the interface.

In addition to the beam focusing ability of the linear phased-array affects caused by changing the curvature of the circular tube material, the length and the width of the element also play an important role in beam propagation. Thus, the effect of the length and width of the element is discussed. Figure 8b,e indicates that the length and width of the element can produce focusing beams at different depth. When the surface curvature of the circular tube was constant, as the length and width of the element increased, the radiation length of the main lobe and of the side lobes increased, and the whole beam also became wider. It can also be seen from the figures that the length of the sound field increased with the increase of the element size, which indicated that the larger the element size, the greater the detection distance would be. In other words, longer and wider elements tended to increase the side lobe amplitude, resulting in more side-leaking of energy and poor beam profiles. Through the above analysis, we can conclude, in the case of consistent transmission frequency, that a large-size probe was suitable for detecting deeper defects. Therefore, it is necessary to determine reasonable length and width of the elements of a linear phased array in practical applications.

The simulation results showed that the tube material with different curvatures needed elements with different sizes to get optimal focusing beams. In future work, we will perform inspection experiments of the defect on the tubular parts using the linear phased-array ultrasonic transducer, and further research will be carried out.

**Figure 9:**
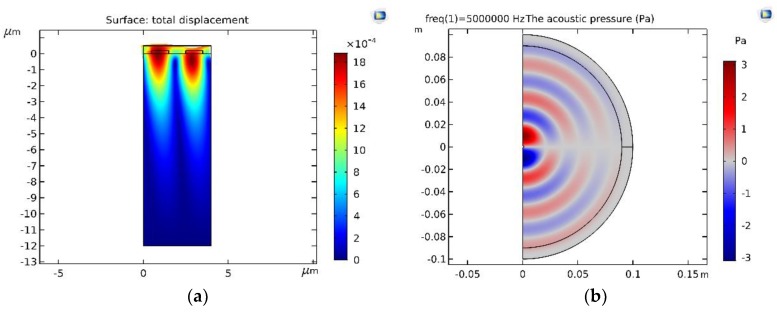
(**a**) Probe simulation by the finite-element method; (**b**) acoustic pressure.

Furthermore, the probe was simulated on the basis of the finite-element method. The acoustic field in the case of a probe with two elements is shown in Figure 9a; Figure 9b is the acoustic pressure. We implemented the finite-element method in COMSOL 5.4 and windows 7. We obtained good agreement between our results and the finite-element results and validated the proposed method by comparing Figure 7a and Figure 9a.

## 6. Experimental Verification

**Figure 10:**
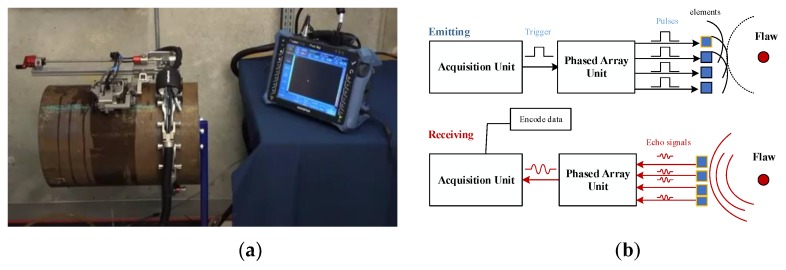
(**a**) Experimental apparatus; (**b**) schematic diagram of the ultrasonic phased array.

The experimental apparatus employed in this work consisted of five components: the excitation and acquisition equipment, the probe, the tested sample, a water injection device, an encoder for recording the UT data, as illustrated in Figure 10a. The excitation and acquisition equipment was an Olympus Omniscan MX2 scanner(16:64). A 5 MHz, 64-element linear transducer array was used in contact with the sample surface with water coupling; the water can be viewed as a wedge conforming to the curvature of the tested circular materials, the tested pipe sample was made of steel with pipe dimeter of 300 mm and wall thickness of 10 mm. The probe was set on the specimen surface and moved slowly along the circumferential direction of the material. The encoder was used to record the UT data and to store the received signals for further processing. The schematic diagram of the ultrasonic phased array is shown in Figure 10b. The parameters for the experiment are listed in Table 2. In this way, the C-scan image was obtained.

**Figure 11:**
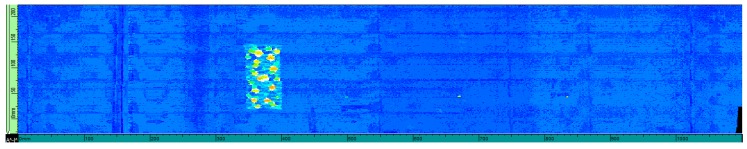
C-scan image of the circular material.

Figure 11 shows the C-scan image obtained by Olympus Omniscan MX2. Image detection data were processed by OmniPC-4.4R4 software for filtering and de-noising. In Figure 11, the C-scan displays color variation: the image color of the defect-free region of the specimen is uniformly blue, while the image color of the defect region is red or yellow. It is clear that the defect could be detected by using the model proposed in the paper.

## 7. Conclusions

In order to meet the strong demand of automated quantitative ultrasonic nondestructive testing for circular tube materials, the MGB, based on the superposition of Gaussian beams, and the expression of the acoustic field from a linear phased-array ultrasonic transducer under condition of a convex interface on the circular tube material were proposed. Ultrasonic phased-array inspection technology was proposed to detect defects in circular tube materials. Acoustic field simulation in the detected circular tube material was carried out. The simulation results indicated that a linear phased-array ultrasonic transducer could produce routine focusing beams in the material, which indicated that the method of ultrasonic phased-array inspection is feasible in circular tube material testing. The simulation results showed that circular tubes with different curvatures and elements with different widths and lengths can produce different focusing beams. Finally, we performed inspection experiments to identify defects in the circular tube material using the proposed linear phased-array ultrasonic transducer and showed that the proposed model allows a reliable, accurate, and detailed defect characterization.

## Figures and Tables

**Table 1:** Multi-Gaussian function superposition coefficients [24].

n	${Α}_{n}$	${Β}_{n}$
1	−2.9716 + 8.6187i	4.1869 − 5.1560i
2	−3.4811 + 0.9687i	3.8398 − 10.8004i
3	−1.3982 − 0.8128i	3.4355 − 16.3582i
4	0.0773 − 0.3303i	2.4618 − 27.7134i
5	2.8798 + 1.6109i	5.4699 + 28.6319i
6	0.1259 − 0.0937i	1.9833 − 33.2885i
7	−0.2641 − 0.6723i	2.9335 − 22.0151i
8	18.019 + 7.8291i	6.3036 + 36.7772i
9	0.0518 + 0.0182i	1.3046 − 38.4650i
10	−16.9438 −9.9384i	6.5889 + 37.0680i
11	0.3708 + 5.4522i	5.5518 + 22.4255i
12	−6.6929 + 4.0722i	5.4013 + 16.7326i
13	−9.3638 − 4.9998i	5.1498 + 11.1249i
14	1.5872 − 15.4212i	4.9665 +5.6855i
15	19.0024 + 3.6850i	4.6296 + 0.3055i

**Table 2:** Experiment parameters.

Parameters	Concrete Setting
Sound Velocity of the Material/(m/s)	5890.0
Laws of the Configuration	Linear at 0^0^
Focus Depth/mm	9.53
First Element	1
Last Element	64
Number of Elements	8
Element Step	1
Sonic Type	Longitudinal Wave
Scanning Mode	Encoder
Imaging Display	A-B, A-C
Maximum Scan Speed/(mm/s)	199.84
Resolution of the Encoder(step/mm)	19.2
